# Ultrastructural Study on the Antibacterial Activity of Artonin E versus Streptomycin against *Staphylococcus aureus* Strains

**DOI:** 10.1371/journal.pone.0128157

**Published:** 2015-06-01

**Authors:** Asdren Zajmi, Najihah Mohd Hashim, Mohamed Ibrahim Noordin, Shaden A. M. Khalifa, Faiqah Ramli, Hapipah Mohd Ali, Hesham R. El-Seedi

**Affiliations:** 1 Department of Pharmacy, Faculty of Medicine, University of Malaya, 50603 Kuala Lumpur, Malaysia; 2 Department of Health Sciences, Faculty of Health and Life Sciences, Management and Science University, 40100 Shah Alam, Malaysia; 3 Center for Natural Products and Drug Discovery (CENAR), Department of Chemistry, Faculty of Science, University of Malaya, 50603 Kuala Lumpur, Malaysia; 4 Department of Experimental Hematology, Karolinska University Hospital, SE-141 86 Stockholm, Sweden; 5 Department of Chemistry, Faculty of Science, University of Malaya, 50603 Kuala Lumpur, Malaysia; 6 Division of Pharmacognosy, Department of Medicinal Chemistry, Uppsala University, Box 574, SE-75 123, Uppsala, Sweden; Indian Institute of Science, INDIA

## Abstract

Staphylococci are facultative anaerobes, perfectly spherical un-encapsulated cocci, with a diameter not exceeding 1 micrometer in diameter. *Staphylococcus aureus* are generally harmless and remain confined to the skin unless they burrow deep into the body, causing life-threatening infections in bones, joints, bloodstream, heart valves and lungs. Among the 20 medically important staphylococci species, *Staphylococcus aureus* is one of the emerging human pathogens. Streptomycin had its highest potency against Staphylococcus infections despite the likelihood of getting a resistant type of staphylococcus strains. Methicillin-resistant *S*. *aureus* (MRSA) is the persister type of *Staphylococcus aureus* and was evolved after decades of antibiotic misuse. Inadequate penetration of the antibiotic is one of the principal factors related to success/failure of the therapy. The active drug needs to reach the bacteria at concentrations necessary to kill or suppress the pathogen's growth. In turn the effectiveness of the treatment relied on the physical properties of *Staphylococcus aureus*. Thus understanding the cell integrity, shape and roughness is crucial to the overall influence of the therapeutic agent on *S*. *aureus* of different origins. Hence our experiments were designed to clarify ultrastructural changes of *S*. *aureus* treated with streptomycin (synthetic compound) in comparison to artonin E (natural compound). In addition to the standard *in vitro* microbial techniques, we used transmission electron microscopy to study the disrupted cell architecture under antibacterial regimen and we correlate this with scanning electron microscopy (SEM) to compare results of both techniques.

## Introduction

*Staphylococcus aureus* has a unique ultrastructural criterion. *S*. *aureus* grows in clusters resembling grapes as they usually do not divide in one plane only. They rather divide in three different horizons where the sister cells change slightly the positions and accordingly the attachment points [1]. This colonization is associated with the formation of extracellular polymeric complex, namely proteins, glycoproteins, glycolipids, polysaccharides [2]. It is generally believed that the bacteria become more resistant and appear more consolidated after encapsulation in an outer membrane at different divisional levels. Due to the structure complexity, the *S*. *aureus* used aerobic fermentation to fulfill the process of metabolic respiration. A process necessitates the cell to use catalase enzymes and release lactic acid without the need of oxygen. In addition to the lactic acid, *S*. *aureus* produce the enzyme coagulase, a catalyzing protein that loosen the body cellular basal membrane thus disrupting the interstitial layers of the tissue and consequently sloughing the infected cells with wider spread of the infection [3].

The traffic of the digestive enzymes from and into the bacteria requires an intact cell organelles, cytosol and plasma membrane. The vacuoles transported from the pathogenic bacteria to the host cells encounter both the biochemical surroundings and the physical barriers [4]. The arrest of the biochemical interaction is claimed to premature termination of protein synthesis due to irreversible asymmetry of the bacterial plasma membrane and cytosol deformability [5]. Malformation of the cell membrane and alterations of the cytoskeleton portrait impacts mainly the cell functionality. The abnormal growth, division, cell dis-configuration such as thickened cells walls, empty cell ghosts and abnormal shapes [2] are amongst the hallmarks to pinpoint whether the bacteria will interact or resist the therapeutic agent within the host cells niche.

Previous research by other teams showed that flavonoid compounds known as artonin E, which was previously isolated from the stem bark of Artocarpus *communis*, have displayed relatively strong antimicrobial activities against Gram-positive, Gram-negative bacteria and *C*. *albicans* [6]. Other groups investigated the ultrastructure changes of *S*. *aureus* under the influence of treatment with the commercially available antibiotics and found that different antibiotics may destroy the *S*. *aureus* architecture and cause variable cell deformity events [7]. Due to the above factors, the current study was designed to test synthetic and natural compounds by transmission electron microscopy (TEM) at subcellular level. We correlate this with SEM results and compare results from both techniques with the standard *in vitro* microbial tests. In this report, artonin E, a prenylated flavonoid, demonstrated a significant antibacterial activity comparable with streptomycin, the conventional antibiotic. The two agents were highly active against *S*. *aureus* and methicillin-resistant *S*. *aureus* (MRSA) isolates with MICs less than 4μg/mL. Artonin E, similar to streptomycin, inhibited the growth of *S*. *aureus* and MRSA as seen by the diffusion disc. Artonin E prevented the construction of the *S*. *aureus* cell wall, arrested the continuation of the plasma membrane and influenced the stability of the bacteria cytoskeleton.

## Results

### Ultrastructure of *S*. *aureus* and MRSA treated with artonin E and streptomycin

**Fig 1:**
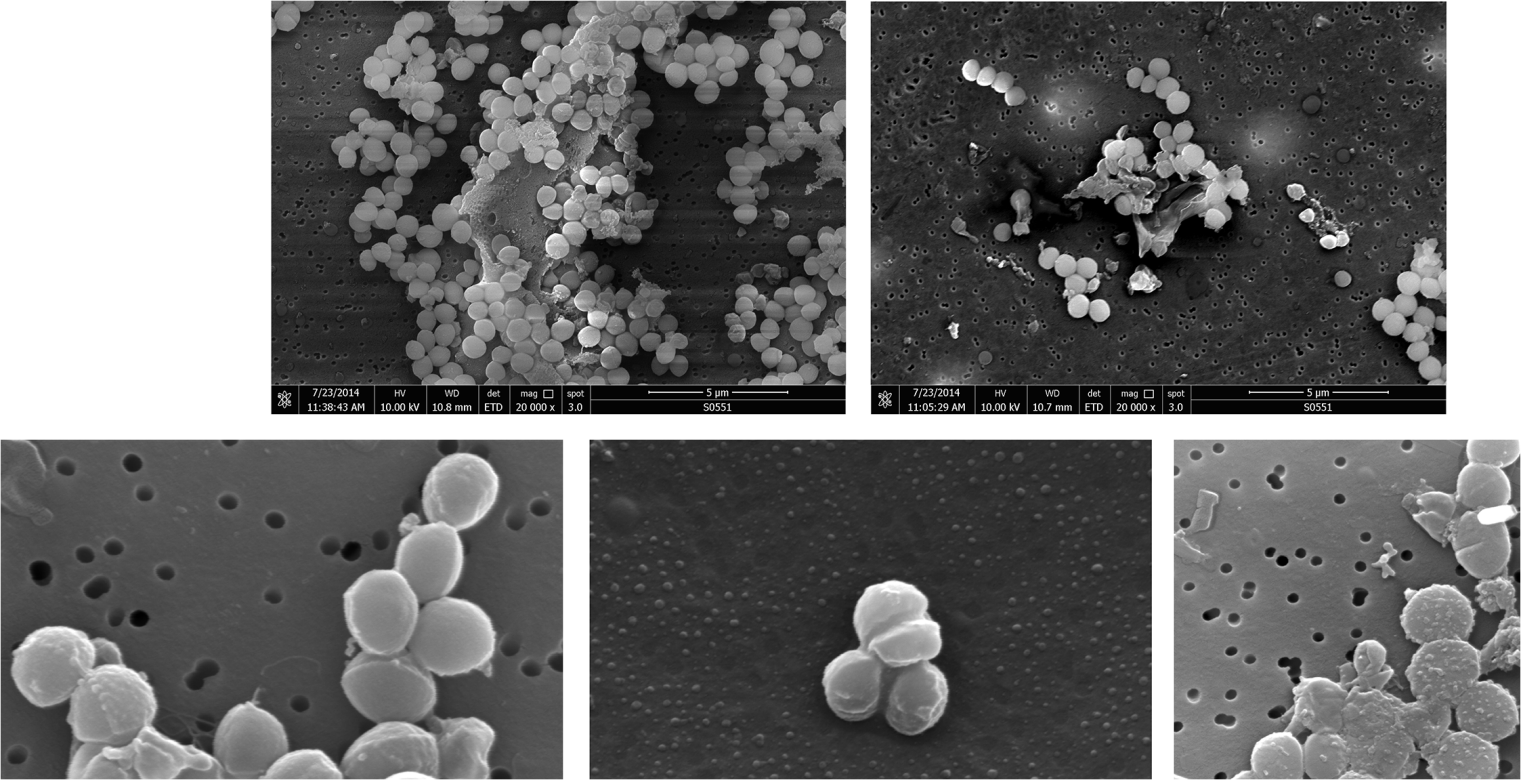
Scanning Electron microscopic analysis. *S*.*aureus* ATCC 25923 (A, B and D) and MRSA ATCC BAA-1720 (C and E) bacteria treated with artonin E (bar = 1mm). *S*.*aureus* ATCC 25923 after 24 hours (A) untreated and (B and D) exposed to the MIC of artonin E. MRSA BAA-1720 after 24 hours (C) untreated and (E) exposed to the MIC of artonin E for 24 h (bar = 1mm). Images are viewed with (A and B) FEI Quanta 650 FEG ESEM and (C, D and E) JEOL JSM 7001F Field Emission Gun SEM.

Representative images of *S*. *aureus* by scanning electron microscopy showed how the bacteria exist as cocci colonies of grape-shape in the control culture with an average size of 0.4 μm (1a). Untreated bacteria were studied as control to justify the observed differences. The SEM micrographs displayed several apparent, distinguished signs of cell envelope damage, including sticky membranes, deep craters, lysed and completely burst cells (1b). SEM pictures also revealed the cytoplasmic turbidity and the irregularity of organelles. Representative images were also provided of the SEM images of *S*. *aureus* treated with artonin E, a compound extracted from natural products. Most of the walls were actually missing, shredder and broken led to distorted shape and focally thickened outer membrane indicating sever damage. The cytoplasm was distributed asymmetrically with eventual budding or septum formation. In addition, the treated Gram-positive cocci exhibit extra-cellular filamentous materials detectable as smoothly curved connections. We observed the difference between control and artonin E-treated culture (Fig 1C–1E).

In the control group cocci-shaped *S*. *aureus* of various sizes were aggregated preferentially within clusters (Fig 1C). The ultrastructural changes were distinguished with SEM as normal appearing cocci could be found in the control in comparison to the cell wall thickening and increase in cell wall roughness of the treated *S*. *aureus* (Fig 1D and 1E). The close-up micrographs of the same culture showed numerous discrete clumps of ruffled membrane, the clumps contained hollow cup-shaped spaces, consistent with the anti-bacterial treatment (Fig 1D). The MRSA cocci, on the other hand were encased in fibrillary sheath in more consolidated-appearing extracellular matrix material. The treated MRSA cocci were seen as decorated with knotty elements, wrinkled cell walls and enmeshed in dense thicker membrane (Fig 1E).

**Fig 2:**
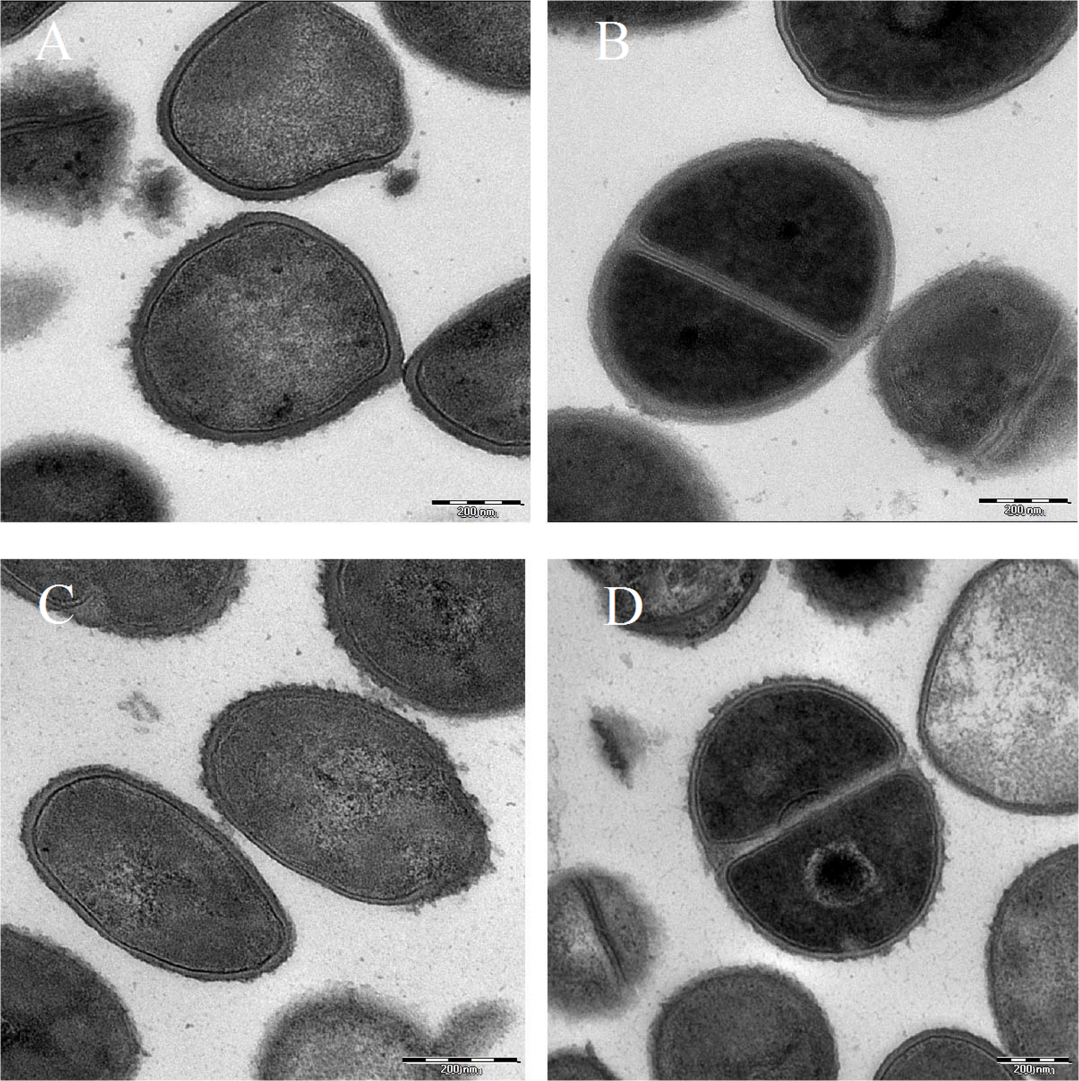
Transmission Electron microscopic analysis. Intracellular density of *S*.*aureus* ATCC 25923 (A) untreated and (C) treated after 24 hours exposed to the MIC of artonin E for 24 hours (bar = 200nm). Intracellular density and cell division of MRSA BAA-1720 (B) untreated and (D) treated after 24 hours exposed to the MIC of artonin E (bar = 200nm).

Representative images of *S*. *aureus* by transmission electron microscopy identified by the presence of bodies, granules, vacuoles and the membrane encapsulated the bacterial cell (Fig 2A and 2B). TEM images of *Staphylococcus aureus* strains treated with artonin E showed the disintegration of bacteria membrane at polar ends as well as cytoplasm leakage. Artonin E treatment for 24 h showed accumulation of the DNA dense material of the *S*.*aureus* ATCC 25923 and thickening of the membrane, whereas obvious lysed membrane was observed in the MRSA ATCC BAA-1720. In contrast, the control (non-treated) cells displayed a highly homogenous intracellular density.

The typical cell wall, outer and cytoplasmic membrane, periplasmic space, and the cytoplasmic content within the electron-dense areas were seen in Fig 2A. In Fig 2B, the division of the cells was characterized by disaggregation of the cytoplasmic matrix, separation of cell membrane and rearrangement into two equal sections. The cytoplasm shows granularity with regions where the DNA aggregations are evident.

The coccoidal shape of *S*. *aureus* was not preserved and deformation/indentations of the cell surface can be observed (Fig 2C and 2D). Intracellular bodies were identified as lamellar aggregates of rough endoplasmic reticulum and other granules. By electron microscopy such granules were large, electron-dense with degranulation and formation of vacuoles following fixation with glutaraldehyde and staining. These observations represent an abnormal intracellular reaction of the treated cells, and probably plenty of phagocytized material (Fig 2C). The bacteria treated with artonin E showed granular deposits, intracytoplasmic coagulation, cytoplasmic retraction, and relative decrease in the DNA material in respect to the cell volume (Fig 2D).

**Fig 3:**
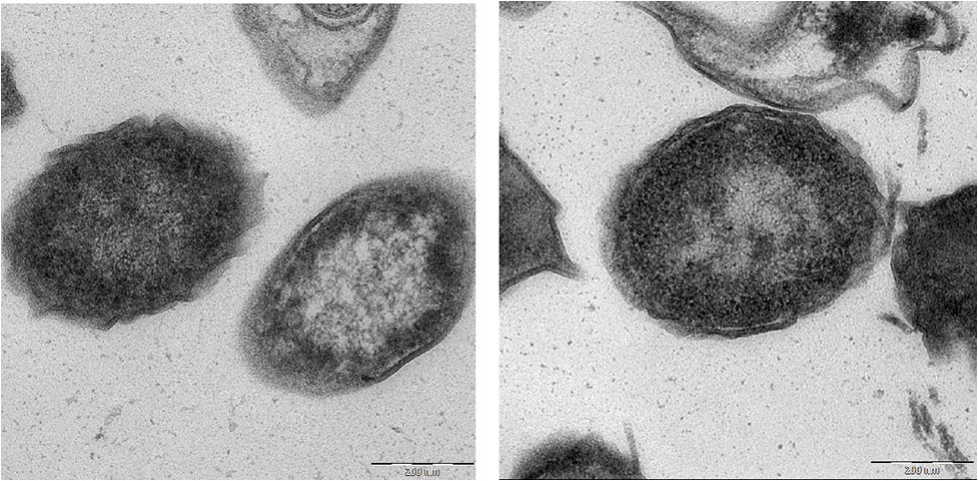
TEM Morphological changes. Ultrastructure and morphological changes of (A) *S*.*aureus* ATCC 25923 and (B) MRSA BAA-1720 exposed to the MIC of artonin E for 24 hours showing peripheral cell surface, hollow formation and cell disintegration (bar = 200nm).

Morphological changes i.e., peripheral cell surface, hollow formation, and cell disintegration were better observed with TEM micrographs. Examples of deformed cells treated with artonin E are shown (Fig 3A and 3B). In Fig 3A, the cell showed wrinkled outer layers, there was no clear distinction between the inner cytoplasmic membrane and the outer cell membrane. Both electron-dense areas at the center of the cell as filamentous densities of several small endocytotic vesicles were seen (Fig 3A). Some of the cells exhibited extra-cellular lamellopodia detected as curved redundant surface. Roughness of the cell surface and the increase of the cell mass were clearly evident (Fig 3B) indicating perforation of the cell wall with subsequent cell deformation.

### Susceptibility of *S*. *aureus* and MRSA to artonin E and streptomycin

Ultimately, the effectiveness of the target compounds should be further addressed by assessing the *in vitro* microbiological activity. A comparison was made between the activity of artonin E and that of streptomycin antibiotic. The antibacterial activity of artonin E and streptomycin was conducted using disc diffusion and dilution methods against *S*. *aureus*. The results show the bactericidal effect of streptomycin and the bacteriostatic effect of artonin E incubated overnight with *S*.*aureus* strains.

Artonin E, at MIC of 3.9 μg/mL, showed strong *in vitro* activity against both *S*. *aureus* isolates (Table 1), including the methicillin-resistant strain.

**Table 1:** Post-antibacterial effects of artonin E and streptomycin in *Staphylococcus aureus* ATCC 25923 and MRSA BAA-1720.

	Inhibition diameter (mm ± SD)	Inhibition diameter (mm ± SD)	MIC (μg/mL)		MIC (μg/mL)	MBC (μg/mL)	MIC (μg/mL)
Compounds	ATCC 25923	ATCC BAA-1720	ATCC 25923	ATCC BAA-1720		ATCC 25923	ATCC BAA-1720
**Artonin E**	13.66 ± 0.57	13.33±0.57	3.9	3.9		7.81	7.81
**Streptomycin (10 μg)**	25.33±0.57	20.0±0	1.95	< 1.0		1.95	1.95

Bacterial growth inhibition was expressed in mm and measured using the disc diffusion assay. Test was performed in triplets. The mean and standard deviations are provided indicating the inhibition diameter. The MIC/MBCs of artonin E were similar for both *S*. *aureus* strains, and comparable MIC and MBCs were obtained using streptomycin. Minimum inhibitory concentration indicates that *S*. *aureus* ATCC 25923 was not grown after exposure to 3.9 μg/mL MIC of artonin E or 1.95 μg/mL of streptomycin, respectively 3.9 μg/mL MIC after the exposure of MRSA BAA-1720 to artonin E or less than 1.0 μg/mL MIC to Streptomycin.

**Fig 4:**
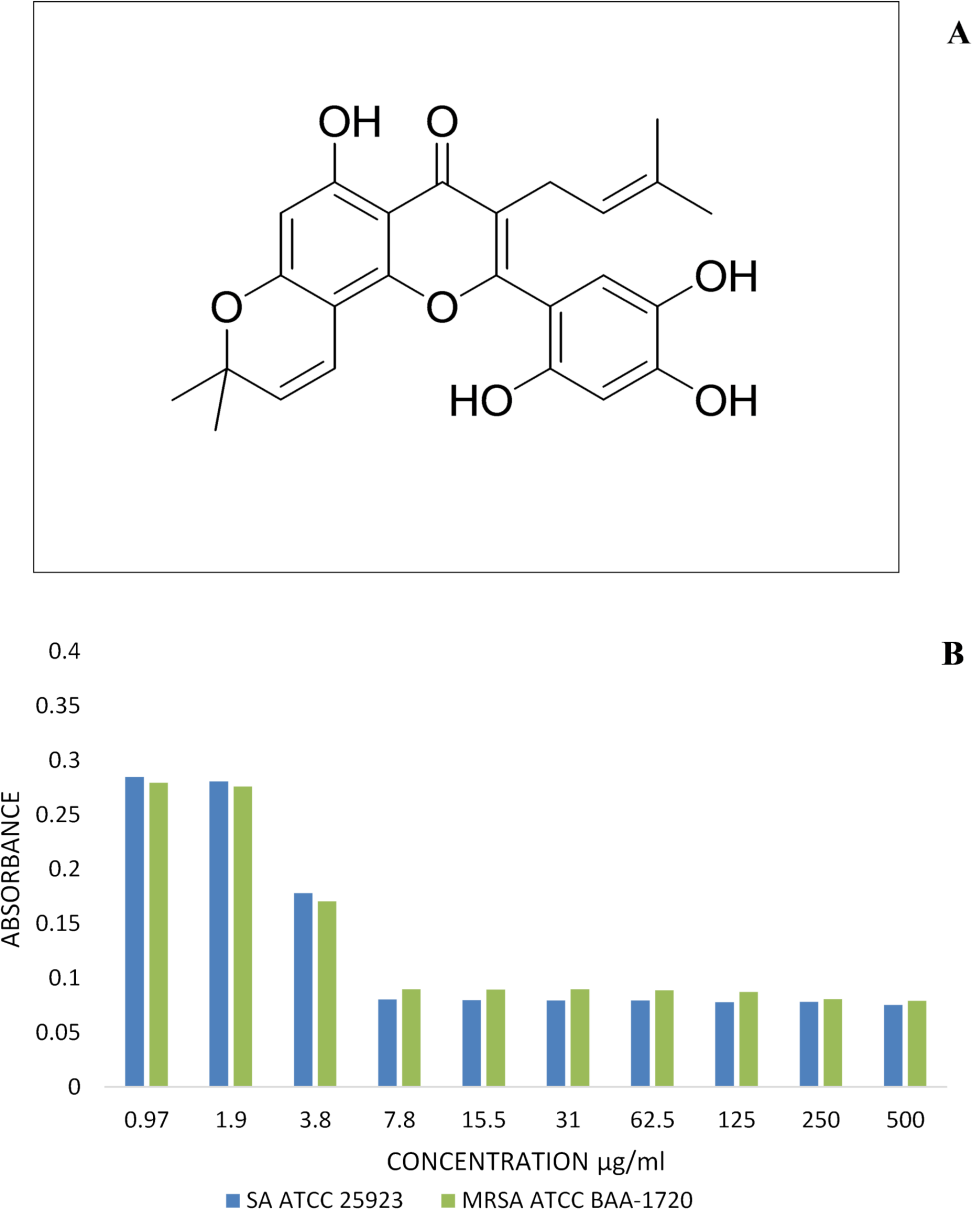
Artonin E bacterial growth and survival. (A) Chemical structure of artonin E. (B) Alamar Blue viability assay showing inhibition of growth of *S*.*aureus* ATCC 25923 and MRSA ATCC BAA-1720 strains in the presence of various concentrations of artonin E incubated in nutrient broth medium at 37°C for 24 hours.

The absorbance obtained in the experiments containing alamar blue dye solution after 4 h of incubation showed decreases of the bacterial cell counts as illustrated in Fig 4. Despite that artonin E was less active than conventional antibiotic streptomycin (MICs, 1.0 to 1.95 μg/mL) against *S*. *aureus* strains. Both *S*. *aureus* ATCC 25923 and MRSA ATCC BAA-1720 isolates treated with artonin E showed a decrease at concentration of 3.9 μg/mL.

**Fig 5:**
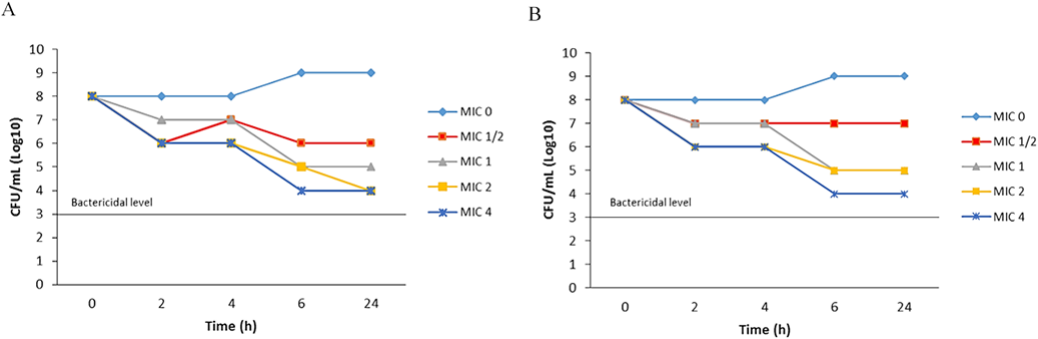
Time killing kinetics. (A) Shown are growth curves of *S*.*aureus* ATCC 25923 and (B) MRSA ATCC BAA-1720 strains bacteria cells grown and evaluated by counting colony forming units (CFU) within different intervals of time (0, 2, 4, 6 and 24 hours) of incubation in MHB II medium at 37°C in the absence or presence of increasing concentrations of artonin E (MIC ½, 1, 2 and 4) added at OD 600 nm of 0.2.

Artonin E affected the eradication of *S*. *aureus* ATCC 25923 and MRSA ATCC BAA-1720 in a concentration-dependent manner (Fig 5A and 5B). The outcome of the time-kill kinetic studies of the two Gram-positive isolates was as summarized in Fig 5. The results showed that artonin E decrease the viable counts of both ATCC 25923 and ATCC BAA-1720 isolates at 4 log10 CFU/mL level. *S*. *aureus* strains used in this study were found susceptible to artonin E (the plant compound) with 13.66±0.57 and 13.33±0.57 mm inhibition zone (Table 1).

The MIC/MBCs of artonin E were similar for both *S*. *aureus* strains, and comparable MIC and MBCs were obtained using streptomycin (Table 1). According to the disc diffusion range, both *S*. *aureus* strains were susceptible to artonin E. Incubation with artonin E and streptomycin resulted in different degrees of killing kinetics for each of the *S*. *aureus* strains. Relatively low concentrations of artonin E were required to inhibit bacterial growth. Streptomycin as well as artonin E showed effects on the viability of *S*. *aureus* and MRSA likely due to the suppression of the bacterial growth as measured by the in vitro tests. Substantial numbers of cells were eradicated at concentrations as low as 3.9 μg/mL. Although the bactericidal effect was not clearly detectable, artonin E changed the bacterial kinetics as assessed by the killer curve, and differences found. The assay was statistically significant and streptomycin showed a higher effect on the cell viability at concentrations as low as < 1.0 μg/mL. Again, significant difference was detectable at higher concentrations. Interestingly, using the disc diffusion and dilution method, a comparable substantial area of killed *S*.*aureus* strains was observed with artonin E similar to streptomycin.

## Discussion

To examine the biological changes at subcellular resolution, we utilized the powerful electron microscopy. Electron microscopies, both transmission and scanning, are predominant for observing the cell structure and its enclosures of singular organelles [8]. In this way, the effect of potential antibacterial agents can be elucidated via the optical sections. In this report, we evaluated the bacterial cellular changes under influence of antibacterial agents and suggested combined strategies to address the suppression or inhibition of cell growth *in vitro*. Additionally, we documented the potential advantage of the TEM and SEM platforms to encompass a number of bacteriostatic/bactericidal properties. The correlation between the SEM visual and TEM signature analysis, allowed us to compare the structure of the plasma membrane, the roughness of the outer surface and the irregularity of the subcellular structures. The independent morphological parameters of the *Staphylococcus aureus* and its organelles described both the susceptibility and sensitivity status of the bacteria to the target compound. Furthermore, Electron microscopy allowed the identification of the visual changes of the cell architecture in correlation to the presence or absence of the targeted molecules at certain doses and thus highlighted the potential effects of the different antibacterial agent.

With the *S*. *aureus*, having a diameter of maximum 1 mm, these combined techniques have the advantage to cross the physical barrier from single-cell imaging to single-cell profiling. The alterations of the *S*. *aureus* outer membrane, visualized by the scanning microscope pinpointed the possibility of plasma membrane leakage and loss of the cell functionality. The plasma membrane discontinuation leads to loss of potassium ions that inhibit respiration, and promote the uptake of propidium iodide [9]. An up-regulation of vital bacteria cell activities has in fact been observed earlier in other antibacterial-treated *S*. *aureus* via transcriptional analysis, in respect to efflux pumping, aerobic energy generation, as well as cell wall and lipid biosynthesis [9]. The hole formation was attributed to the alterations in the cytoskeletal matrix and the connections between indentation appearance and the detachment of the outer glycoprotein complex. Additionally, the increase of the ribonucleic condensation may be a factor for the heavy abundance of the ribonucleus and nucleotides. This basically explained the ultrastructural roughness of the nucleus and the DNA coagulation. We also found superficial protein structure rearrangement that with the SEM visual analysis correlated well with the presence of mesosomes. Similarly, the non-treated bacteria were seen primarily as cell clusters, connecting and intermingling with interstitial fibers that anchored their connection to each other and to the surface. The micrographs obtained from non-treated bacteria, showed the envelope of the cocci in extracellular material and seemed as every individual bacteria compile copious extracellular-related materials. We conclude by the lyses of the bacteria and the variations in size and shape, that the bacteria were exposed to difference in the osmotic pressure.

The 20th century witnessed the discovery of antibiotic broad spectrum and it sounded like the golden age for medicine, till the resistant bacteria evolved in the late 70^th^ [10]. The resistant bacteria are seriously difficult to cure by available antibiotics and have been a leading cause of lethal staphylococcus cases (Doggrell, 2005). The advantage of one category over the other varied considerably over time and between clinics taking into consideration the advantages and disadvantages. So far, evidence is scarce to support how particular bacterial strains respond to specific antibacterial agent and the resistance remains an ongoing problem. The ability of antibacterial agents to cross the plasma membrane barrier is an important consideration for the future antibacterial applications. In resistant cases, it seems like antibiotics do not efficiently penetrate the bacteria; therefore, some infections require higher doses of the drugs [11]. Availability of active drug is affected by the degree of cell membrane binding. Higher doses than those reflected by *in vitro* data may be necessary clinically. Potentially adverse clinical consequences may result from the high doses due to the rapid lytic action of antibacterial agents. Intensive lytic action could result in the production of increased cell wall fragments, which intensify the immune response.

Ultimately, the susceptibility of staphylococcus strains to new compounds has to be examined thoroughly in order to evaluate their potential pharmaceutical implications. The target compounds should be studied in relevance to the bacterial growth conditions, density, test duration extent of reduction in bacterial numbers and ultrastructural variations [12]. Accordingly, various *in vitro* microbiological techniques coupled with T- and S- electron microscopy were employed to investigate the *in vitro* bacteriostatic/bactericidal action of the tested compound (Artonin E)

Disc diffusion is a standard method to estimate the bacterial viability in response to different classes of antibiotics. The first order of the inhibition zone represents the accepted undulate nature of the antibacterial effect relating to the diffusion of the compounds in correlation to the penetration of the growing bacteria on the underlying agar. The minimum bactericidal concentration (MBC) is one of the indicators to show the effect on certain bacteria in the presence of definite antibiotic concentrations. The amount of antibacterial that inhibits visible growth (inhibitory phase) of the microorganism is called the (MIC). Time-kill curves have been used to determine the kinetics of bacterial killing in vitro. The selection of the killer curve is guided by the biological properties of the bacteria [13]. Its main advantage being that qualitative visualization are accompanied by the corresponding quantitative data of the bacterial growth under different circumstances, which allows for further measurements and analysis. This method has been successful in estimating the lowest concentration of an antibacterial agent that either totally prevents or suppress growth results in the initial inoculum in culture. This microtiter testing is useful in distinguishing whether bacterial killing is concentration and/or time dependent. The MIC and MBC activities of the artonin E emphasize that the compound is active against Gram-positive bacteria. The artonin E *in vitro* dilution against both *S*. *aureus* isolates displayed MIC of 3.9 μg/mL and MBC of 7.81μg/mL. The time killing studies showed that artonin E exhibited strong effects against *S*.*aureus* at four times concentrations of MIC, most likely due to bacteriostatic activity.

*In vitro* antimicrobial assays data provide information on the potential action of natural product compound as antibacterial agents, but this is only few of many factors necessary to predict a favorable clinical outcome. Hence, the visual resolution of subcellular levels has made the T- and S- electron microscopy a potential platform of bacterial single-cell investigation. The ultrastructural changes discussed here are not visible using a traditional light microscope [14]. The results seen by the biological tests combined with TEM and SEM analyses of *S*. *aureus* might give valuable information about the status of the bacteria with feasible treatment regimens. The techniques open the field of microbiology to new possibilities, including screening of new antimicrobials. Additional experiments with *in vivo* models would be useful for assessing the mechanism of action and to provide the appropriate scientific justification.

## Materials and Methods

### Materials and Reagents

Nutrient Agar (DIFCO, Becton Dickinson) and Nutrient Broth (DIFCO, Becton Dickinson) were used for disc diffusion as well as MIC and MBC. Muller Hinton II broth cation adjusted (Becton Dickson) and Muller Hinton Agar was used for time killing assay. AlamarBlue cell viability reagent kit (alamarBlue, Invitrogen) was used for bacteria viability assay. The 96-well microplates (NEST, Nest Biotech Co. Ltd) were used for MIC, MBC and time kill curve. SEM specimen stubs (SPI Supplies, Structure Probe, Inc., PA, USA) were used for the embedding of the bacteria sample for the SEM processing. Sodium Phosphate Monobasic (Na2HPO4, anhydrous, Fisher Scientific) and Glutaraldehyde reagent (E.M. Grade, SPI Supplies, Structure Probe, Inc., USA) were used for SEM and TEM.

### Organisms

The two *S*. *aureus* isolates used in this study, namely *Staphylococcus aureus* (ATCC 25923) and methicillin-resistant *Staphylococcus aureus* (ATCC BAA-1720), were obtained from the American Type Culture Collection (ATCC). Inoculate of the *S*.*aureus* organisms were prepared using the colony suspension method. Colonies picked from 24 hours old cultures grown on nutrient agar (NA) were cultured in nutrient broth (NB) for 24 hour at 37°C.

### Transmission Electron Microscope

The bacterial cells suspension were diluted in nutrient broth (NB) to 0.5 McFar-land’s standard and treated accordingly with MIC of artonin E for 24 h at 37°C. These cells were consequently fixed with 4% glutaraldehyde, then post-fix for 15 hour in 1% osmium tetroxide. Graded ethanol series were used to dehydrate the samples prior to washing with propylene oxide-epon. Ultrathin 100 nm sectioning was prepared and stained with 3% uranyl acetate. Samples were viewed using Zeiss 912 Omega (Oberkochen, Germany) microscope.

### Scanning Electron Microscope

Bacteria for SEM samples were prepared, as explained previously. After fixing the bacteria, they were washed and re-suspended in water and post-fixed with 0.1% OsO4. The treated cell suspension was then put on a membrane filter (pore size 0.1 μm). Subsequently, the samples were dehydrated with graded series of ethanol (30–95%) and acetone mixture series (3:1, 1:1, 1:3) on membrane filters. Critical point drying process was conducted for 1 hour and coated with gold and carbon in sputter coater. Microscopy was performed with a JEOL JSM 7001F Field Emission Gun Scanning Electron Microscope (FEG SEM) (Japan).

### Disc-Diffusion

Petri plates (9 cm) (NEST) were prepared with 20 ml of a base layer of Nutrient Agar (DIFCO, Becton Dickinson, USA) prior to seeding of bacterial suspension (10^6^ CFU/mL). For each 6 mm diameter discs we used 10 μl of 1 mg/mL of compound. Discs containing standard antibiotic (streptomycin) was used as positive control. The diameter of the inhibition zones was measured and evaluated in millimeters after 24 h of incubation at 37°C. All tests were performed in triplicate and the antibacterial activity was expressed as the mean of inhibition diameters in millimeter produced (±standard deviation).

### MIC and MBC

The standard broth micro dilution method was used to determine the minimum inhibitory concentrations (MIC) and minimum bactericidal concentration (MBC) for the plant compound and streptomycin, using standardized test bacteria (OD600 nm) as modified from the early described protocol [15] with some modifications. Using a 96-well microtitre plate (NEST, Nest Biotech Co. Ltd) and Nutrient Broth (DIFCO, Becton Dickinson, USA), bacteria and compound were distributed into each well. After incubation, control wells 12 A-H were loaded with nutrient broth without any addition, whereas wells 11 A-H were loaded with broth and bacteria without treatment. Quantification of the optical density in each well was carried out using a micro plate reader (TECAN, Infinite M200PRO, Switzerland). Wells showing zero growth on the plate were referred to as MBC.

### ABA

AlamarBlue cell viability reagent (alamarBlue, Invitrogen) was used to assess cell viability by simply adding 10% of the reagent to the final volume followed by 4 hours in-cubation at 37°C. ABA was performed in flat, clear-bottomed, 96-well microplates (NEST, Nest Biotech Co. Ltd). Determination of cell viability was done through plot-ting normalized absorbance at 570 nm, versus compound concentration per well. The high absorbance values were attributed to the increased total metabolic activity of the cells.

### Time Kill Curve

Time kill curve was conducted following the standard guidelines [16]. The bacterial suspensions for inoculation were prepared from fresh cultures on Muller Hinton II broth cation adjusted. The isolated colonies were suspended in sterile saline and the culture turbidity was adjusted to 0.5 McFarland’s standard. The suspension was diluted further to get required inoculum (i.e. 5 X 10^7^ CFU/mL). The compounds were dissolved using 100% DMSO for stock preparation and subsequent dilutions were made.

The Mueller Hinton II broth was used with *S*. *aureus* ATCC 25923 and MRSA ATCC BAA-1720 isolates. The inoculum was diluted (1/100) into 10 mL MHB to give 5 X 10^5^ CFU/mL. The compounds were added to the respective flasks at selected concentration. No addition to the flasks was considered as negative control ‘0 X MIC’.

Five time points (0, 2, 4, 6 and 24 hours) were selected for sampling following centrifugation at 37°C at 110 rpm. The samples were serially diluted and re-cultured in the Muller Hinton agar then incubated overnight at 37°C. To determine log CFU values, the platted graph were defined as to bactericidal or bacteriostatic based on the CFU reduction at various times intervals. The compounds were said to be bactericidal if it exhibits ≥ 3 log CFU reduction and bacteriostatic if they exhibited < 3 log reduction.
